# Trends in Atrial Fibrillation Ablation Utilization Following 2022 CMS Reimbursement Reduction: A Nationwide Analysis

**DOI:** 10.1111/jce.70102

**Published:** 2025-09-09

**Authors:** Pranav Puri, Daniel Moe, Ashraf Alzahrani, Ghazaleh Goldar, Brian Olshansky, Peter Farjo, Paari Dominic

**Affiliations:** ^1^ University of Iowa Hospitals and Clinics Iowa City Iowa USA

## Abstract

**Background:**

In 2022, the Centers for Medicare & Medicaid Services (CMS) implemented a bundled payment policy that substantially reduced reimbursement for atrial fibrillation (AF) ablation procedures, raising concerns about potential effects on utilization and procedural complexity.

**Objective:**

To evaluate national trends in AF ablation volumes, reimbursement, and procedural complexity following the 2022 CMS reimbursement change.

**Methods:**

Using the Medicare Physician and Other Practitioners by Geography and Service Dataset from 2016 to 2023, we identified pulmonary vein isolation (PVI) procedures using CPT 93656 and additional ablations beyond PVI using CPT 93657. Reimbursement rates were inflation‐adjusted to 2023 dollars. Joinpoint regression was used to assess trends before and after the 2022 policy change.

**Results:**

From 2016 to 2023, national AF ablation volume increased with an average annual percent change (AAPC) of 11.7% (95% CI, 8.2%–14.0%; *p* < 0.01). Following the 2022 CMS bundled payment policy, inflation‐adjusted reimbursement declined by 27.7% in 2022 and by a further 20% in 2023 (slope change *p* = 0.01). Despite this, total AF ablation volume rose by 24.3% between 2021 and 2023, and procedures involving additional ablation beyond PVI increased by 42.4% over the same period. Joinpoint regression showed no significant inflection in PVI or additional ablation volume trends following the policy change (*p* = 0.87 and *p* = 0.97, respectively).

**Conclusions:**

Despite significant reimbursement reductions following Medicare's 2022 policy change, electrophysiologists continued to perform increasing numbers of AF ablations, including procedures with additional ablations beyond PVI.

## 1 Introduction

Catheter ablation has become a cornerstone in the management of atrial fibrillation (AF). Large, randomized clinical trials such as CASTLE‐AF and CABANA have demonstrated the superiority of early ablation in improving symptom control, quality of life, and long‐term cardiovascular events [1, 2]. As a result, utilization of catheter ablation has steadily increased in recent years.

In 2022, the Centers for Medicare & Medicaid Services (CMS) implemented a significant policy change under the Medicare Physician Fee Schedule (MPFS) [3]. AF ablation, 3D mapping, and intracardiac echocardiography (ICE) were bundled into a single procedural code, resulting in a 25% reduction in work relative value units (RVUs) compared to billing each separately. This policy shift has raised concerns about potential downstream effects on physician practice patterns, procedural volumes, and access to care. This study uses national Medicare data to describe trends in AF ablation volumes and reimbursements following the 2022 CMS policy change.

## 2 Methods

We used CMS's Medicare Physician and Other Practitioners by Geography and Service Dataset to extract data on AF ablation procedures, including associated 3D mapping and ICE. Current Procedural Terminology (CPT) codes for these procedures were identified for the years 2016 through 2023. Specifically, CPT code 93656 was used to identify pulmonary vein isolation (PVI) procedures, and CPT code 93657 was used to identify additional ablations beyond PVI. National average reimbursement rates for AF ablation were calculated for each year before 2022 by summing rates for PVI (CPT code 93656) to 3D mapping (CPT code 93613) and ICE (CPT code 93621). Post‐2022 rates were determined based on the new bundled payment structure. Only facility‐based procedures (defined as procedures not done in the office setting) were included in this analysis to ensure consistency and accuracy of reimbursement rates. Reimbursement values were inflation‐adjusted to 2023 dollars using the CPI. The number of electrophysiologists (EPs) was estimated by tabulating the number of distinct National Provider Identifiers (NPIs) who billed Medicare for CPT code 93656.

Joinpoint regression was performed to identify changes in trends over time. Our Joinpoint regression analysis focused on detecting significant changes in overall trends rather than individual year‐to‐year variations. A *p*‐value of < 0.05 was considered statistically significant.

## 3 Results

**Figure 1:**
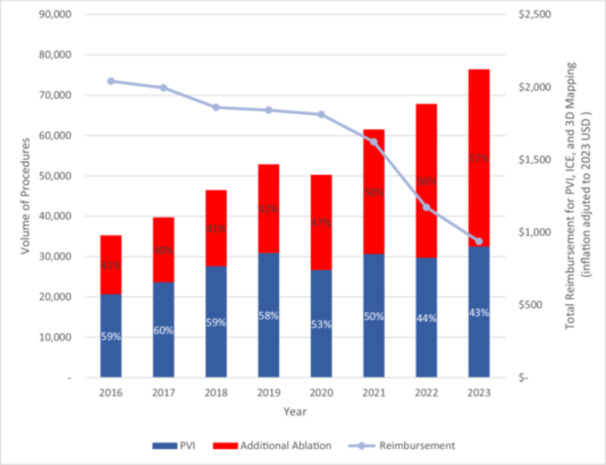
Impact of 2022 CMS reimbursement policy on AF ablation procedure complexity and volume.

Between 2016 and 2023, AF ablation volumes increased with an annual percent change (APC) of 11.7% (95% CI 8.2%–14.0%, *p* < 0.01). Average inflation‐adjusted reimbursement for AF ablation, including 3D mapping and ICE, decreased by 27.7%, from $1623 in 2021 to $1172 in 2022 and a further 20% to $938 in 2023. Despite the significant reimbursement reduction in 2022, AF ablation volumes increased from 61 495 in 2021 to 67 845 in 2022 (+10.3%) and further to 76 401 in 2023 (+12.6%). From 2021 to 2022, the volume of procedures involving additional ablations beyond PVI (CPT code 93657) increased by 23.6%, from 30 829 to 38 115 in 2022 and by a further 15.2% to 43 908 in 2023 (Figure 1). The number of EPs billing Medicare for PVI grew from 1033 in 2016 to 2813 in 2023.

Indexed to this workforce, PVI volume per EP decreased from 20.0 in 2016 to 11.6 in 2023 (overall APC −3.7%, *p* < 0.01). Additional ablation volume per EP increased from 14.1 in 2016 to 17.9 in 2021, peaked at 20.5 in 2022, and declined to 15.6 in 2023 (overall APC 4.3%, *p* = 0.04). Join‐point regression with 2022 specified as the breakpoint confirmed no significant slope change for PVI (pre‐ vs. post‐2022 AAPC +10.9% vs. +12.6%, *p* = 0.87) or additional ablation volume (+17.0% vs. +17.0%, *p* = 0.97), but demonstrated a significantly significant decline in reimbursement (−4.5% vs. −20.0%, *p* = 0.01) and growth in the number of EPs billing for AF ablation (+10.7% vs. +50.8%, *p* = 0.01) after the policy change.

## 4 Discussion

Despite a 27.7% reduction in reimbursement under the 2022 CMS policy change and a further 20% decline in 2023, AF ablation volumes continued to grow at a comparable rate to prior years. Similarly, the proportion of procedures involving additional interventions beyond PVI continued to increase at a similar rate as before the policy change. Indexed to the expanding EP workforce, PVI volumes per EP fell over the study period, while additional ablations beyond PVI per EP increased. These trends are consistent with prior research on the effects of bundled payment schemes for hip fracture and lumbar fusion surgeries, which were associated with increased procedural volumes and a shift towards higher cost procedures for more complex cases [4, 5]. The growth in additional interventions beyond PVI could also reflect delays in treatment related to the COVID‐19 pandemic and higher volumes of progression from paroxysmal to persistent AF.

## 5 Limitations

This study is limited by its retrospective design and reliance on Medicare billing data, which do not capture patient‐level clinical characteristics, physician decision‐making, or institutional factors that may have influenced trends in ablation volumes. For example, the data used in this analysis do not allow for comparisons in the use of cryoballoon versus radiofrequency ablations, which may affect additional ablation volumes. In addition, our analysis did not include CPT 93655 in the main procedural volume trends because it can be billed alongside multiple primary ablation codes, and our data set does not delineate the specific combinations used. This exclusion may underrepresent the total volume of complex ablation procedures but avoids conflating distinct procedural categories. Additionally, our data set does not differentiate between initial and repeat ablation procedures. Similarly, there may be a lag between reimbursement cuts and their full impact on practice patterns; therefore, our findings may not capture delayed behavioral responses to the 2022 policy change and 2023 reimbursement reductions.

## 6 Conclusions

In conclusion, despite a 42.2% reduction in inflation‐adjusted reimbursement between 2021 and 2023, EPs continued to perform increasing numbers of AF ablations, including procedures with additional ablations beyond PVI. Future studies should evaluate whether this trend reflects shifts in disease burden, physician decision‐making, or latent impacts on patient access and procedural appropriateness.

## Ethics Statement

This study utilized CMS's Medicare Physician and Other Practitioners by Geography and Service Dataset, which is publicly available data and does not contain personally identifiable information. The study adhered to ethical guidelines by ensuring responsible use of the data.

## Conflicts of Interest

The authors declare no conflicts of interest.

## Data Availability

The data that support the findings of this study are available in CMS Data at https://data.cms.gov/. These data were derived from the following resources available in the public domain: Center for Medicare and Medicaid Services, https://data.cms.gov/.
